# Supplementary material for: Pepsinogen C Interacts with IQGAP1 to Inhibit the Metastasis of Gastric Cancer Cells by Suppressing Rho-GTPase Pathway
Source: Cancers (Basel). 2024 May 8;16(10):1796. doi: 10.3390/cancers16101796 (PMC11120368; doi:10.3390/cancers16101796)

Original WB figures of Figure 4A

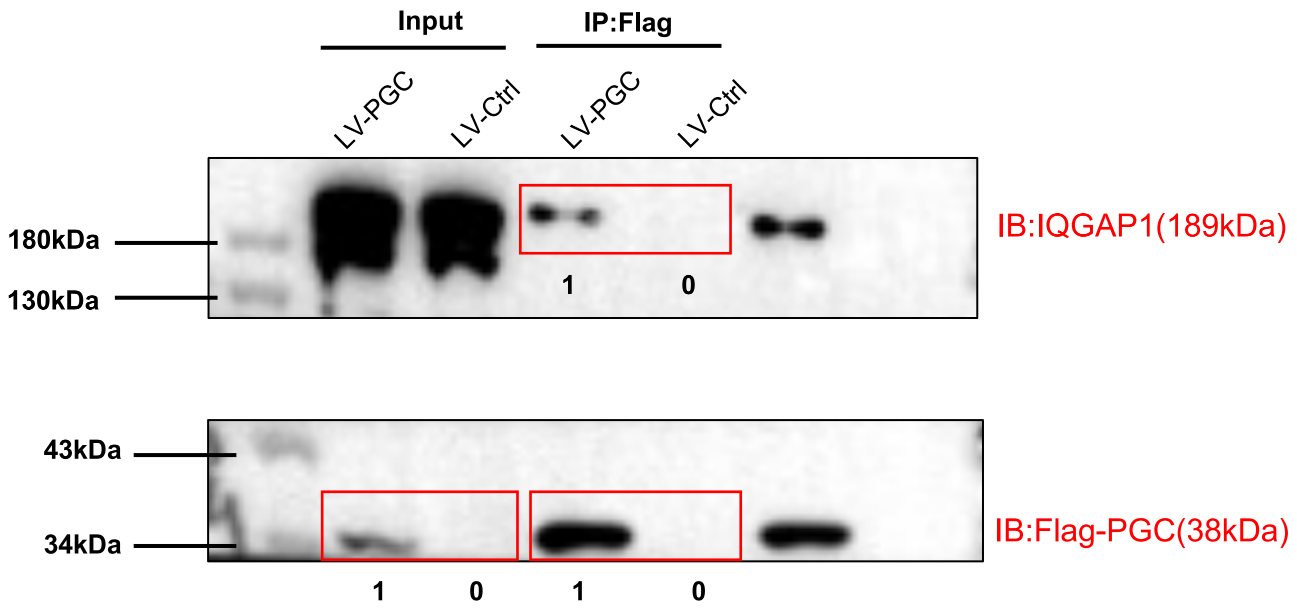

Original WB figures of Figure 4B

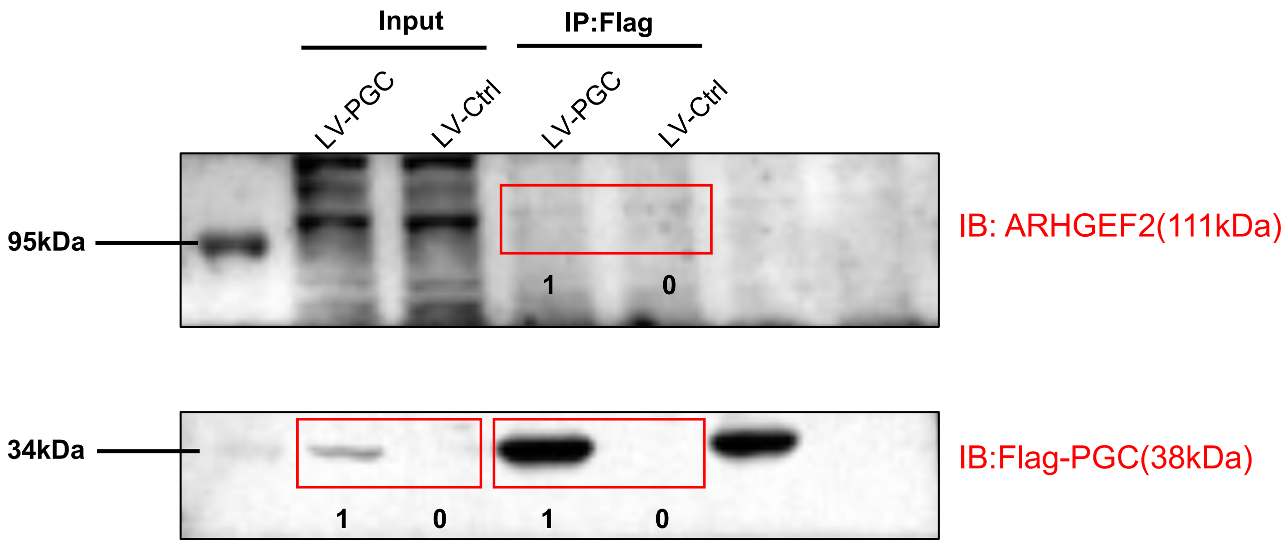

Original WB figures of Figure 4C

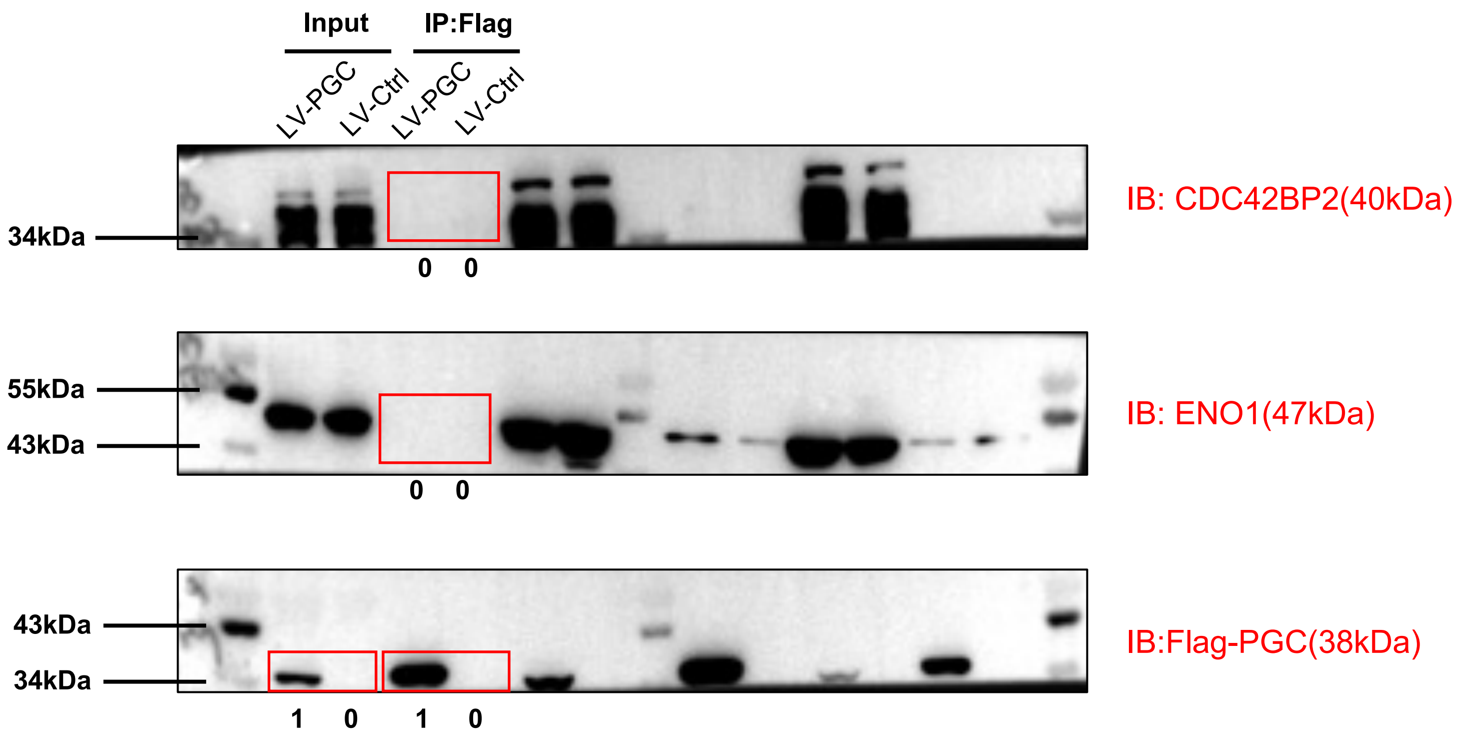

Original WB figures of Figure 4D

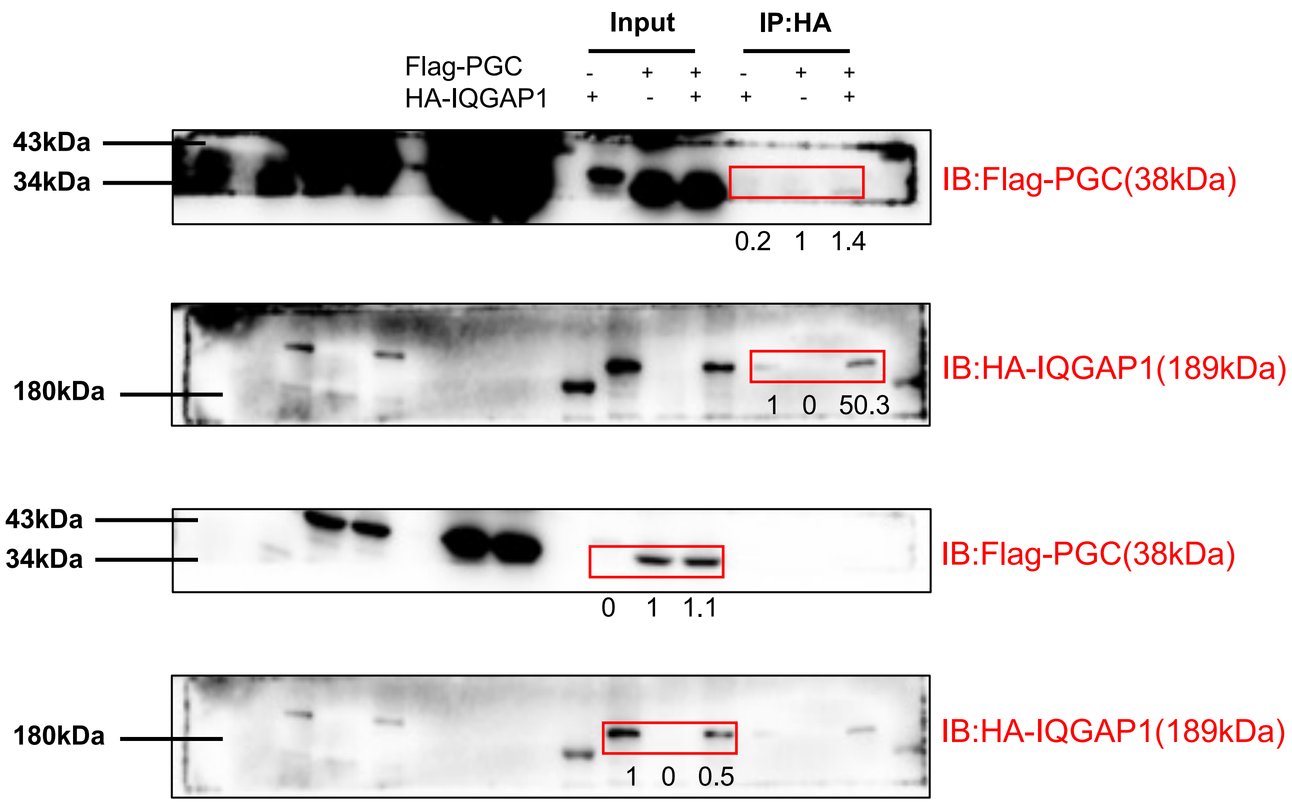

Original WB figures of Figure 4G

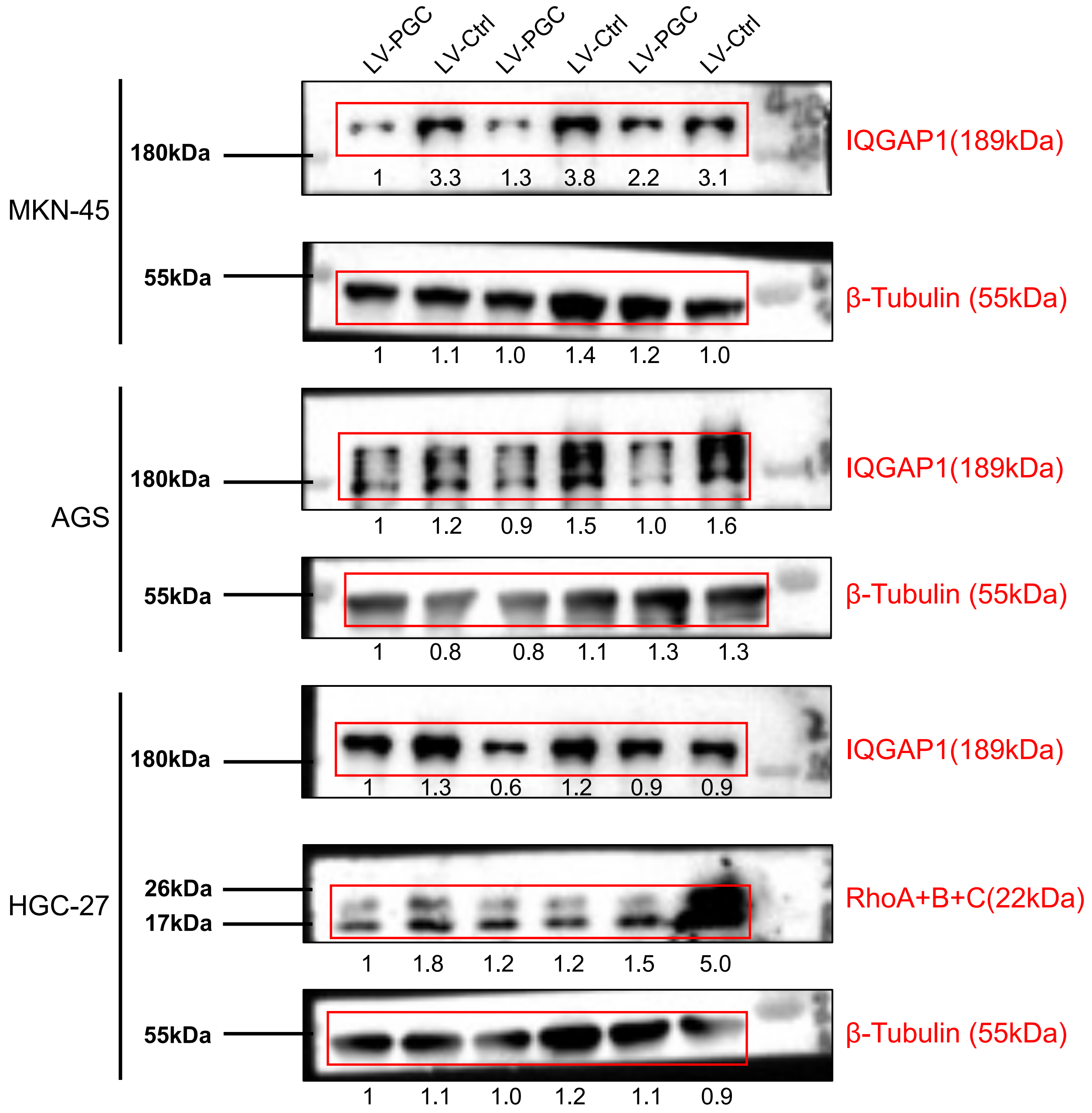

Original WB figures of Figure 4I

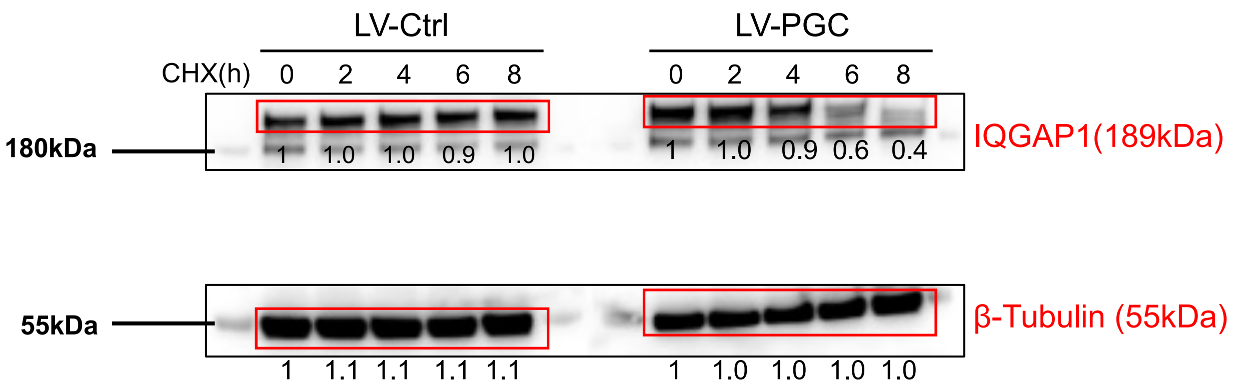

Original WB figures of Figure 4J

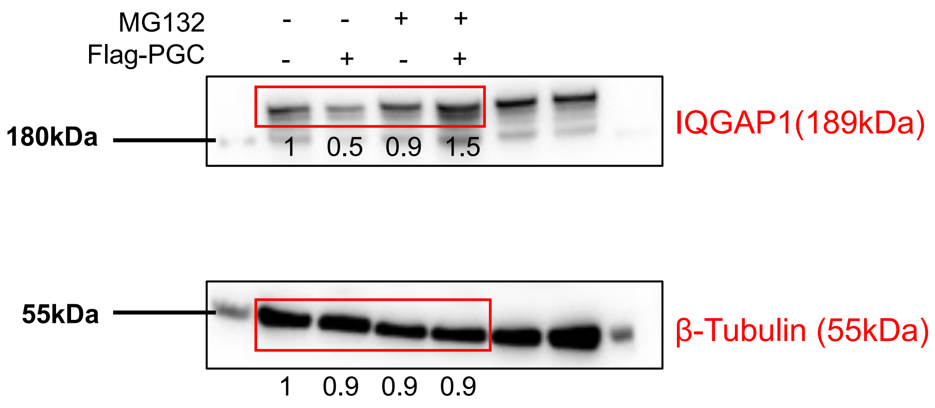

Original WB figures of Figure 5G

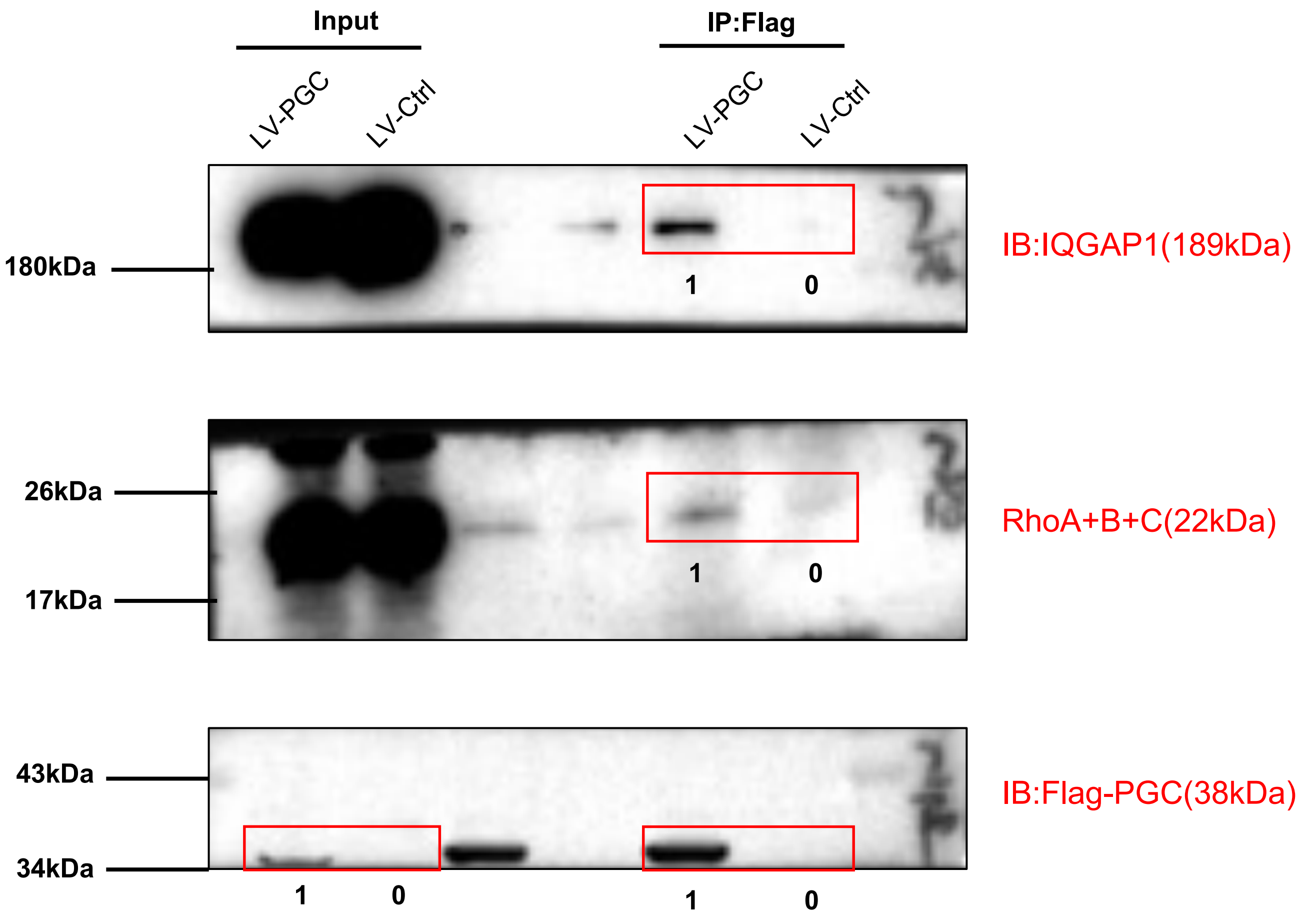

Original WB figures of Figure S1D

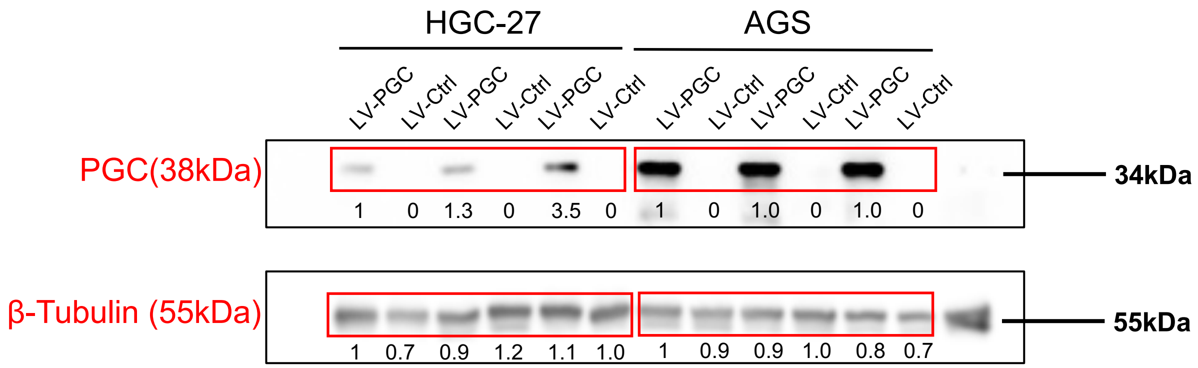

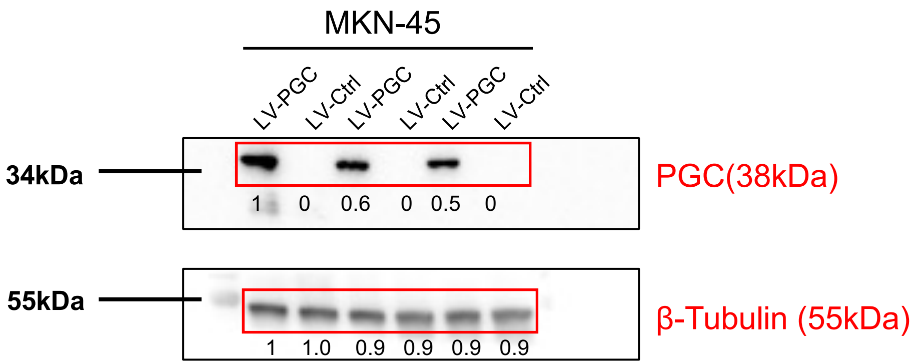

Supplement: Supplementary file 1 [file cancers-16-01796-s001.zip › Original WB Figures0508.pdf]
