# Supplementary material for: Pepsinogen C Interacts with IQGAP1 to Inhibit the Metastasis of Gastric Cancer Cells by Suppressing Rho-GTPase Pathway
Source: Cancers (Basel). 2024 May 8;16(10):1796. doi: 10.3390/cancers16101796 (PMC11120368; doi:10.3390/cancers16101796)
Supplement: Supplementary file 1 [file cancers-16-01796-s001.zip › Supplementary Materials-Figures.pdf]

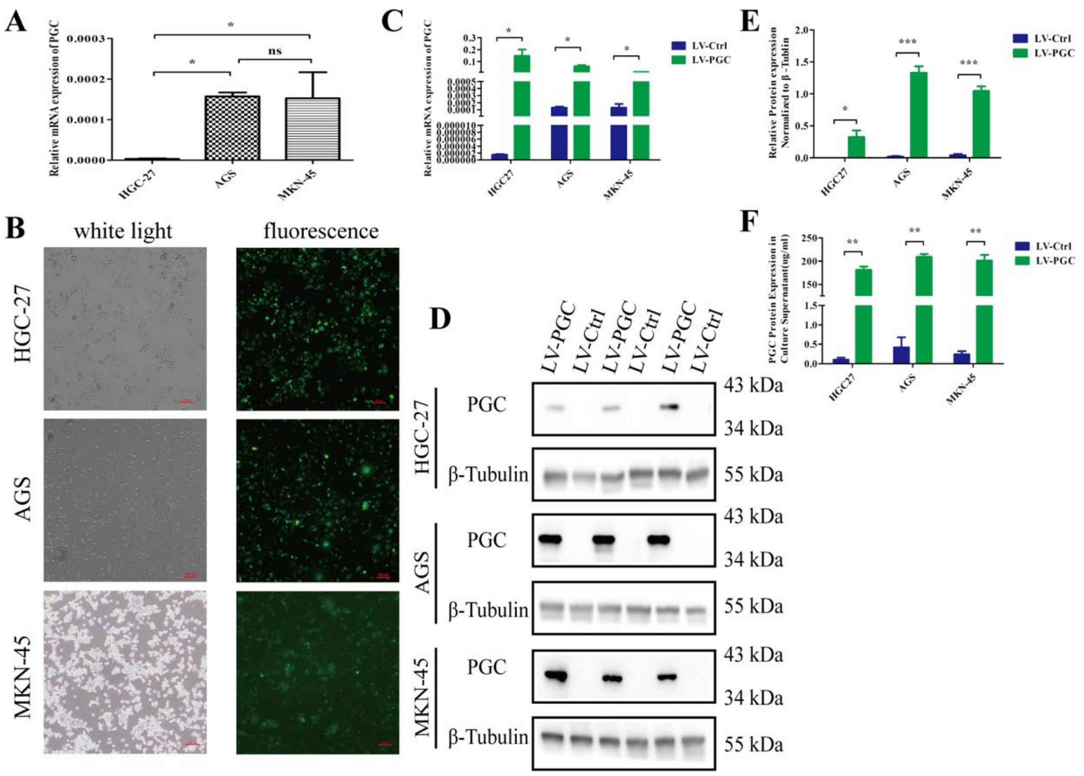

Figure S1. The overexpression efficiency of PGC. **A** Background expression of PGC in the three GC cell lines. **B** Fluorescence microscopy observation of PGC-lentivirus infection efficiency in HGC-27, AGS, and MKN-45 cells (100×). **C-E** qRT-PCR (**C**), WB (**D-E**), and ELISA (**F**) were used to determine the overexpression efficiency of PGC. The results suggested that the PGC overexpression model was successfully constructed (*p <0.05, **p < 0.01, ***p< 0.001, ns, p>0.05).

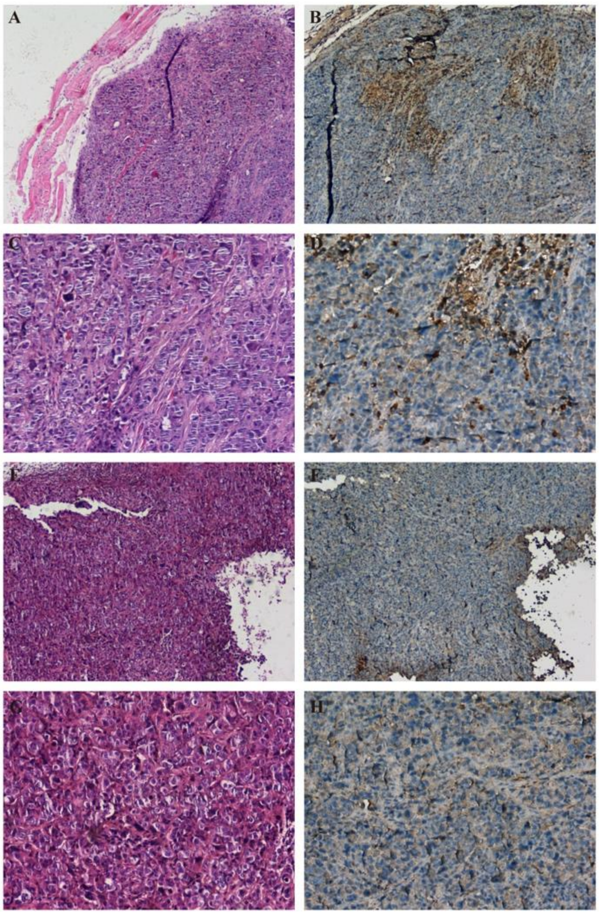

Figure S2. HE staining and PGC immunohistochemical staining of xenograft tissues. **A-D** The expression of PGC in the xenografts of the stably expressing PGC group (LV-PGC) was positive. **E-H** The expression of PGC in the xenografts of the negative control vector group (LV-Ctrl) was negative.

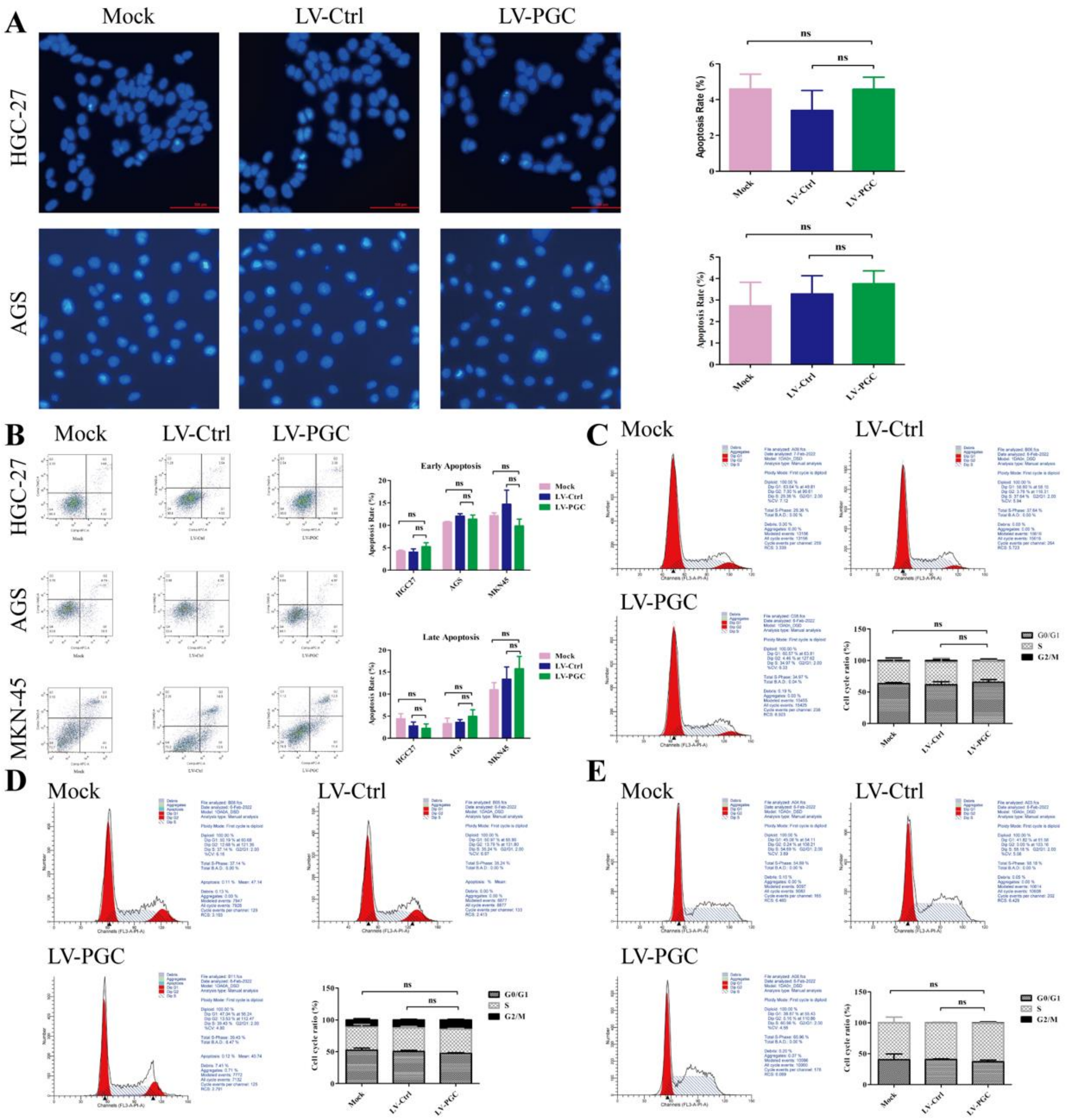

Figure S3. PGC is not correlated with GC cells apoptosis or cell cycle progression. **A-B** Cell apoptosis was measured after the transfection of HGC-27, AGS, and MKN-45 cells with LV-PGC and LV-Ctrl by using Hoechst staining assays (**A**) and flow cytometry (**B**). The results suggested that PGC did not affect GC cell apoptosis. **C-E** Cell cycle progression was evaluated after the transfection of HGC-27 and AGS cells with LV-PGC and LV-Ctrl by using flow cytometry. The results showed that PGC did not affect the phase distribution of GC cells.

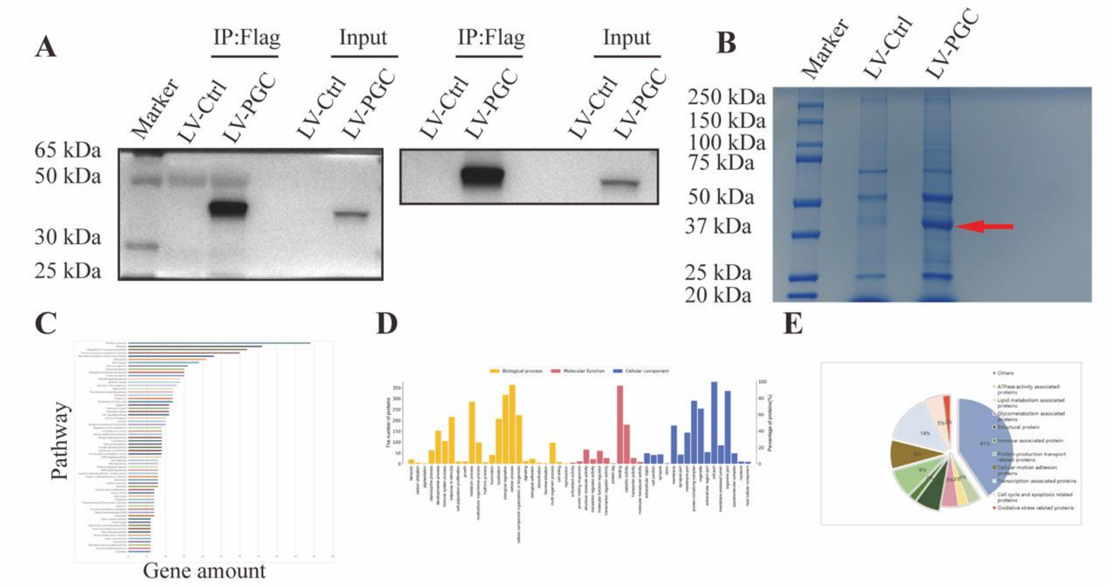

Figure S4. Identification of PGC-targeted proteins in GC by Co-IP and LC/MS. **A** Verification of the precipitation of FLAG-PGC and FLAG fusion magnetic beads. **B** Coomassie brilliatnt blue staining verified that FLAG fusion magnetic beads could co-precipitate to PGC-interacting proteins. **C-D** GO and KEGG analysis of all of the PGC-interacting proteins. **E** The proteins with specific functions are displayed in the diagram and were annotated by using the UniProt database.
