# Supplementary material for: Pepsinogen C Interacts with IQGAP1 to Inhibit the Metastasis of Gastric Cancer Cells by Suppressing Rho-GTPase Pathway
Source: Cancers (Basel). 2024 May 8;16(10):1796. doi: 10.3390/cancers16101796 (PMC11120368; doi:10.3390/cancers16101796)
Supplement: Supplementary file 1 [file cancers-16-01796-s001.zip › Supplementary Materials-Tables.pdf]

Supplementary Table S1. Sequences of primers used in this manuscript

Gene		Primer Sequence
PGC	Forward	5'-CTCTGTCCGTGGATGAGGC-3'
	Reverse	5'-CCAAAGACAACCGCTCCC-3'
E-cadherin	Forward	5'-TTGACTTGAGCCAGCTGCACA-3'
	Reverse	5'-GGAACCGCTTCCTTCATAGTC-3'
N-cadherin	Forward	5'-GGCATACACCATG CCATCTT-3'
	Reverse	5'-GTGCATGAAGGACAGCCTCT-3'
Vimentin	Forward	5'-TGACCGCTTCGCCAACTACAT-3'
	Reverse	5'-TCCCGCATCTCCTCCTCGTA-3'
Snail	Forward	5'-AGGCAGCTATTTCAGCCTCC-3'
	Reverse	5'-CACATCGGTCAGACCAGAGC-3'
Slug	Forward	5'-GCTACCCAATGGCCTCTCTC-3'
	Reverse	5'-CTTCAATGGCATGGGGGTCT-3'
Twist	Forward	5'-GCCAGGTACATCGACTTCCTCT-3'
	Reverse	5'-TCCATCCTCCAGACCGAGAAGG-3'
Fibronectin	Forward	5'-CCCACCGTCTCAACATGCTTAG-3'
	Reverse	5'-CTCGGCTTCCTCCATAACAAGTAC-3'
MMP2	Forward	5'-TACAGGATCATTGGCTACACACC-3'
	Reverse	5'-GGTCACATCGCTCCAGACT-3'
MMP9	Forward	5'-TGTACCGCTATGGTTACACTCG-3'
	Reverse	5'-GGCAGGGACAGTTGCTTCT-3'
MIST1	Forward	5'-AGGCCCTGCGTGAAGTCAT-3'
	Reverse	5'-CCGTCAGCGATTTGATGTAGTTC-3'
ATP4A	Forward	5'-TCACCACCGACGACAATCTG-3'
	Reverse	5'-GGTGACGACAACCACAGCAA-3'

ATP4B	Forward	5'-GTCAGTTCCCTGCCAAATCATC-3'
	Reverse	5'-GGAGTTCGCACACTGACAACAC-3'
TFF1	Forward	5'-GCCCTCCCAGTGTGCAAAT-3'
	Reverse	5'-CGTCGATGGTATTAGGATAGAAGCA-3'
TFF3	Forward	5'-CCCTGCAGGAAGCAGAATGC-3'
	Reverse	5'-CGAAGAACTGTCCTCGGGTG-3'
MUC5AC	Forward	5'-CAGCACAACCCCTGTTTCAAA-3'
	Reverse	5'-GCGCACAGAGGATGACAGT-3'
MUC6	Forward	5'-CAGCAGGAGGAGATCACGTTCAAG-3'
	Reverse	5'-GTGGGTGTTTTCCTGTCTGTCATC-3'
GAST	Forward	5'-CCCAGCTCTGCAGACGAGAT-3'
	Reverse	5'-GCCAGAGCCAGTGCAAAGAT-3'
GKN1	Forward	5'-CAGTCAACCCAAACAAAGTCGAT-3'
	Reverse	5'-CCCACGACACATGTTTGCAA-3'
NEUROG3	Forward	5'-GACGGAGTCGGCGAAAGAA-3'
	Reverse	5'-CCGAGTTGAGGTTGTGCATTC-3'
CD44	Forward	5'-TGATGGCACCCGCTATGTC-3'
	Reverse	5'-AGCCGCTGCTCACGTCAT-3'
CD133	Forward	5'-TCCCATAAAGCTGGACCCATT-3'
	Reverse	5'-ATCACGCGGCTGTACCACAT-3'
Bmi-1	Forward	5'-CGTGTATTGTTCGTTACCTGGAGAC-3'
	Reverse	5'-CATTGGCAGCATCAGCAGAAGG-3'
SOX2	Forward	5'-AGTCTCAAGCGACGAAAAA-3'
	Reverse	5'-GCAGAAGCCTCCTCTTGAA-3'
SOX9	Forward	5'-GCACTCATAATATGGCATCCTTCA-3'
	Reverse	5'-AAACACGAACACAAACCAAAGCTT-3'

VIL1	Forward	5'-CTACAGGAAGAAAACCTGGTCATCA-3'
	Reverse	5'-GCGCCCAGTCTTGTTGGA-3'
4-Oct	Forward	5'-GGTGGAGGAAGCTGACAACA-3'
	Reverse	5'-TGATCTGCTGCAGTGTGGGA-3'
IQGAP1	Forward	5'-TTGTTGCTCCTGATGCCTTTG-3'
	Reverse	5'-GCAATGGAGCCCAGATTTCG-3'
β-actin	Forward	5'-ATGTGGCCGAGGACTTTGATT-3'
	Reverse	5'-AGTGGGGTGGCTTTTAGGATG-3'

Supplementary Table S2. The expression of differentiation and stemness markers in gastric cancer cells with PGC overexpression

	**HGC27**	**AGS**	**MKN45**
**Differentiation marker**			
MIST1	upregulated	not significant	not significant
ATP4A	not significant	not significant	not significant
ATP4B	not significant	not significant	not significant
TFF1	upregulated	downregulated	not significant
TFF3	not significant	not significant	not significant
MUC5AC	upregulated	not significant	not significant
MUC6	upregulated	not significant	not significant
GAST	upregulated	not significant	not significant
GKNI	upregulated	not significant	upregulated
NEUROG3	not significant	not significant	not significant
**Stemness marker**			
CD44	downregulated	downregulated	not significant
CD133	downregulated	downregulated	downregulated
Bmi1	not significant	not significant	downregulated
SOX2	downregulated	downregulated	downregulated
SOX9	downregulated	not significant	downregulated
VIL1	downregulated	downregulated	not significant
OCT4	not significant	downregulated	not significant
