# Supplementary material for: Pepsinogen C Interacts with IQGAP1 to Inhibit the Metastasis of Gastric Cancer Cells by Suppressing Rho-GTPase Pathway
Source: Cancers (Basel). 2024 May 8;16(10):1796. doi: 10.3390/cancers16101796 (PMC11120368; doi:10.3390/cancers16101796)
Supplement: Supplementary file 1 [file cancers-16-01796-s001.zip › Supplementary Materials.pdf]

Supplementary Materials

Pepsinogen C Interacts with IQGAP1 to Inhibit the Metastasis of Gastric Cancer Cells by Suppressing Rho-GTPase pathway

Supplementary Materials and methods

Cell culture and lentivirus infection

HGC-27, AGS, and MKN-45 cell lines were purchased from the Institute of Basic Medical Science Chinese Academy of Medical Sciences (Beijing, China). HGC-27 and MKN-45 cells were cultured in RPMI 1640 medium (HyClone) with 10% fetal bovine serum (FBS, Biological Industries), and AGS cells were cultured in Ham's F-12 (Procell) supplemented with 10% FBS. All cell lines were checked free of mycoplasma contamination and authenticated with STR profiling. PGC-FLAG-EGFP lentiviral vector and the negative control vector were purchased from Shanghai Genechem Company (Shanghai, China) and transfected cells according to the manufacturer's instructions. Stably transduced cell lines were obtained by selection with Puromycin for PGC and Neomycin for IQ-domain GTPase-activating protein 1 (IQGAP1). After 7 days of antibiotic screening, the cells were collected to determine the effects of overexpression in subsequent experiments.

RNA extraction and quantitative real-time PCR

According to the manufacturer's protocol, TRIzol (Invitrogen) was used to extract RNA from tissues and cells. The RNA with A_{260}/A_{280} ratio between 1.7 and 2.1 was converted into cDNA using PrimeScript RT Master Mix (Takara). The quantitative real-time PCR (qRT-PCR) assay was conducted by using the SYBR Green Master Mix Kit (Takara) under the following conditions: 95℃ for 30s and then 40 cycles of 95℃ for 10s, 56℃ for 20s, and 72℃ for 30s. The relative transcription level of target genes were calculated by the relative quantitative method ($2^{-\Delta Ct}$ method), andβ-actin was used as an internal reference for calibration. All primer sequences used in this study are shown in Supplementary Table 1.

Protein extraction and western blot assay

Cells were lysed on ice using cell lysis buffer (Solarbio) containing a mixture of protease inhibitor, phosphatase inhibitor and PMSF (MCE). Protein concentrations were determined using a BCA assay kit (Solarbio) following the manufacturer's protocol. Approximately 20 μg of protein from each sample was separated by sodium dodecyl sulfate-polyacrylamide gel electrophoresis (SDS-PAGE) and then transferred to the polyvinylidene fluoride membrane (Millipore). After treatment with high-efficiency blocking buffer for western blot (WB), the membrane was incubated with the primary antibody overnight at 4℃ and then with the corresponding secondary antibody for 2h at room temperature. Finally, ECL detection reagent (Beyotime) was used to visualizing the protein bands. The primary antibodies used in this study were showed as follows: anti-PGC (1:1000; #ab255826, Abcam), anti-IQGAP1 (1:1000; #ab133490, Abcam), anti-ARHGEF2 (1:2000, #ab201687, Abcam), anti-CDC42BPA (1:1000, #ab146566, Abcam), anti-ENO1 (1:1000, #227978, Abcam), anti-Rho (1:1000, #ab188103, Abcam), anti-FLAG (1:1000, AF519, Beyotime Biotechnology), and anti-HA (1:1000, AH158, Beyotime Biotechnology). β-tubulin (1:1000, abs830032, Absin) and GAPDH (1:1000, abs830030, Absin) were used as loading controls.

Enzyme-linked immunosorbent assay

The collected culture supernatant of GC cells centrifuged at 12000 rpm at 4℃ for 10 min to remove cell debris. PGC expression level in the cell culture supernatant was determined by using a pepsinogen II enzyme-linked immunosorbent assay (ELISA) kit as per the instructions (BioHit). The absorbance value at 450 nm was measured using a spectrophotometer (Thermo Scientific, USA) after the reaction was terminated, and then the PGC protein concentration in the sample was calculated according to the formula.

5-Ethynyl-2'-deoxyuridine incorporation assay

GC cells were seeded in 24-well plates for the determination of DNA synthesis and cell proliferation using 5-Ethynyl-2'-deoxyuridine (EdU) assay kit (Beyotime). When cells reached 60%-70% confluence, preheated complete culture medium

containing EdU solution was added to the plates and incubated for 2 h. Then, after cells were treated with fixative, permeabilization solution, and wash buffer, freshly prepared Click-iT reaction solution was added, and incubated at room temperature in the dark for 30 min. Finally, Hoechst 33342 was used to stain the nuclei. Representative images were observed and obtained using a fluorescence microscope (Olympus, Japan). According to the manufacturer's protocol, DNA synthesis was calculated by the ratio of EdU-positive cells (red) to Hoechst-positive cells (blue).

Cell Counting Kit-8 assay

GC cells were seeded in a 96-well plates (1×10^4 per well) and observed for 72 hours, and cell viability was tested using Cell Counting Kit-8 (CCK-8) solution (10 μl/well) every 24 hours (MCE). After incubation at 37°C for 1.5-h, the absorbance value at 450 nm was measured by microplate reader.

Cell apoptosis assay

GC cells were digested with EDTA-free trypsin and adjusted to 5×10^5 per sample in 500 μl binding buffer and then incubated at room temperature for 15 min after the addition of 5 ul Annexin V-APC and 5 μl 7-AAD (KeyGEN BioTECH) in the dark. Finally, cell apoptosis was determined via a flow cytometer (BD, USA) and the apoptosis rate was calculated using FlowJo software.

Hoechst staining assay

Seeded GC cells were stained with 10× Hoechst 33342 (Beyotime). The apoptosis rate was evaluated using a fluorescence microscope (Olympus, Japan).

Cell cycle assay

GC cells were cleaned 3 times with precooled PBS and then fixed overnight in 70%-75% ethanol at -20°C. Ethanol was removed after fixation and washed with PBS. For this assay, cells were then stained with PI/RNase Staining Buffer (Thermo Fisher) for 30 min in the dark, and determined by flow cytometry (BD, USA). The cell cycle phase distribution was analyzed by ModFit software.

Sphere formation assay

GC cells were inoculated in the ultra-low adhesion 24-well plates (Corning) at a density of 3000 cells per well and incubated with RPMI 1640 medium containing 2%

B27 (Thermo Fisher), 20 ng/ml bFGF (Solarbio) and 20 ng/ml EGF (MCE) for 7 days. To calculate the sphere formation efficiency, spheroids those between 40 and 100 μm in size were counted using an inverted microscope (Olympus, Japan).

Transmission electron microscopy (TEM) assay

GC cell samples were fixed in 2.5% glutaraldehyde at 4°C for 24 h. Subsequently, these samples were immobilized with 1% osmic acid, dehydrated with ethanol and acetone, and embedded in 812 embedding agent. Finally, sectioned samples were stained with lead citrate, and then observed by a transmission electron microscopy (TEM) (HITACHI, Japan) to obtain representative images.

Wound healing assay

GC cells were seeded and cultured in the 6-well plate until confluence and then the cell layer was scratched with a 100-μl sterile pipette tip. After being scratched (recorded as 0 h), the cells were incubated in serum-free medium for 24 h (recorded as 24 h). The cell migration distance was captured and measured using a universal microscope (Olympus, Japan) and Image J.

Transwell assay

To assess cell migration, 3×10^4 GC cells suspended in serum-free medium were seeded in the upper chamber, while RPMI 1640 with 10% FBS was added into the lower chamber. In the invasion assay, Matrigel (BD) was precoated on the upper membrane surface of a Transwell chamber and then 5×10^4 GC cells were inoculated in the chamber. After being incubated for 24 h (AGS cells) and for 48 h (HGC-27 and MKN-45 cells), cells that migrated or invaded the surface of lower membrane were fixed with 4% paraformaldehyde and stained with 0.1% crystal violet solution (Beyotime) for visualization. The migrated or invaded cells were captured and counted under an inverted microscope (Olympus, Japan).

Co-immunoprecipitation and liquid chromatography-mass spectrometry

Co-immunoprecipitation (Co-IP) assay was performed using an immunoprecipitation kit following the manufacturer's guidelines (Beyotime). Total protein of AGS cells stably transfected with PGC (FLAG-tagged) overexpression vector or empty vector was extracted by using ice-cold cell lysis buffer that

supplemented with protease inhibitor cocktail. Then the protein complexes were precipitated with 3× FLAG magnetic beads overnight at 4°C. Beads with protein complexes were washed in PBST buffer and replaced by 3× FLAG peptide. Subsequently, loading buffer was added into the final supernatant and protein electrophoresis was conducted. The SDS-PAGE gels were stained with Coomassie bright blue solution to identify the target protein bands and digested into peptides with trypsin. Finally, the polypeptides were analyzed using an LC-MS instrument (Thermo Scientific) and the original data of each sample were checked against the Protein Database via PD/MASCOT software to obtain the protein spectrum results.

Co-IP of PGC and IQGAP1 in GC cells

According to the above method, total protein of AGS cells that stably transfected with PGC (FLAG-tagged) overexpression vector or IQGAP1 (HA-tagged) overexpression vector was extracted. Lysate (at least 1 mg protein) and magnetic beads fused with FLAG label or HA label was incubated overnight at 4°C. WB was used to determine the expression of PGC and IQGAP1 protein.

Cycloheximide and MG132 assays

Cycloheximide (CHX) chase assay was performed to determine the half-life of IQGAP1 protein. GC cells stably transfected with PGC overexpression vector or empty vector were treated with the protein synthesis inhibitor CHX (100 μM/ml) for 2 h, 4 h, 6 h, and 8 h. GC cells overexpressing PGC protein and the corresponding control cells were treated with the proteasome inhibitor MG132 (20 μM/ml) for 6h to block the proteasomal degradation of IQGAP1 protein prior to cell lysing with lysis buffer. Then, total protein of each time point was extracted and the expression level of IQGAP1 protein was determined by WB.

Immunofluorescence staining

GC cells were seeded onto the slide and cultured overnight. After discarding the culture medium and washing three times with PBS, cells were fixed with 4% paraformaldehyde and treated with Triton X-100. Cells were blocked with donkey serum at 37°C for 30min, and then incubated with IQGAP1 antibodies (1:100; ab86064, Abcam) overnight at 4°C. The next day, after being washed three times with

PBST, cells were incubated with fluorescent secondary antibody (Thermo Fisher) and then stained with DAPI. Finally, protein fluorescence pictures were captured and evaluated using a fluorescence microscope.

Hematoxylin and eosin staining

After fixation with 4% paraformaldehyde for 1 week, the tissue samples of the in vivo animal experiments were then embedded in paraffin and sliced into 4-μm-thick sections. The tissues sections were dewaxed and rehydrated and stained with hematoxylin and eosin (H&E) for histopathological examination. Finally, representative images were obtained using a microscope.

In vivo animal experiments

Specific pathogen-free grade male BALA/C nude mice, weighting 16-18 g and aged 4 to 5 weeks, were purchased from SPF Biotechnology Company (Beijing, China). All in vivo animal experimental protocols were approved by the Animal Care and Use Committee of the First Hospital of China Medical University.

First, MKN-45 cells stably overexpressing PGC and the corresponding control cells were established via lentivirus infection. For tumorigenicity assays, 5×10^6 MKN-45 cells (LV-PGC and LV-Ctrl) suspended in 100 μl normal saline (NS) solution were injected into the left flank of each nude mouse (7 mice per group). The xenografts size was measured every 3 days with a manual caliper and calculated according to the following formula: tumor volume = (length × width^2)/2. After 20 days of GC cell injection, the mice were sacrificed for xenografts subcutaneous tumor collection and measurement. For tumor growth assays, 5×10^6 wild-type MKN-45 cells suspended in 100 μl NS were injected into the right flank of each nude mouse. When the subcutaneous xenograft tumor grows to around 80-100 mm^3, the tumor-bearing mice were divided into control and experimental group (5 mice per group) with similar starting tumor volumes. Control and experimental group mice were intratumoral injected with human PGC active protein (hPGCp) and NS respectively every 3 days, 5 times in total. According to the protocol, tumor volume was measured and calculated every 5 days. Mice were sacrificed for tumor harvesting after twenty days of the first injection of PGC protein.

A peritoneal dissemination assay was performed via intraperitoneal injection. Briefly, 2×10^6 MKN-45 cells (LV-PGC and LV-Ctrl) suspended in 100 μl NS solution were injected into the peritoneal cavity of each nude mouse (5 mice per group). Peritoneal metastasis mice were sacrificed and analyzed 20 days after the injection. All of the metastatic tumors and organs were resected for H&E staining.

Bioinformatics analysis

The differential expression levels of PGC and IQGAP1 mRNA as well as the expression correlation in 408 GC and 211 normal control tissues were analyzed using the GEPIA2 database and GTEx databases [16]. The correlation between PGC and IQGAP1 mRNA expression level and the GC patients survival was analyzed via the Kaplan-Meier Plotter database [17]. In addition, we also analyzed the PGC and IQGAP1 protein expression differences in 80 GC and paired normal tissues using the CPTAC database [18, 19].

Patients and tissue specimens

20 paired pathologically confirmed GC and adjacent normal tissues collected from the First Hospital of China Medical University were used to evaluate the PGC and IQGAP1 mRNA expression differences and the expression correlation in GC patients. The protocol was approved by the First Hospital of China Medical University Ethics Committee, and written informed consent was obtained from all patients before operation.

Statistical analysis

In this study, statistical analyses were carried out by SPSS 23.0 software and GraphPad Prism 5. Results are presented as means ± standard error of the mean (SEM). Two-tailed Student's t-test was used for normally distributed data, while rank-sum test was applied for skewed distributed data. A p value < 0.05 was considered statistically significant.
